# M. D. and D. D. S.

**Published:** 1872-07

**Authors:** H. L. Ambler


### M. D. and D. D. S.

BY H. L. AMBLER, D. D. S., M. D.

Liberality toward our brothers in the practice of the different professions is a virtue to be commended, for the more we understand of them and their specialties the better we will be able to appreciate both, as it is true that thorough acquaintance with the principles of an art or science doubles the pleasure we receive from it. All arts being founded on science, shows that they are dependent upon each other, the relation between the two being that of offspring and parent. Many of the useful arts have been the issues of necessity, and, of course, are the first to originate; but combined with experience, they are sure to progress, if it is in a slower manner than the fine arts. Dental surgery is both a science and an art. As a science, it investigates, analyzes and defines the principles upon which the profession is founded; as an art, it enables us to apply these principles, and in the most proper manner to exhibit our special skill. If we store our minds with knowledge, it may some time lead to a practical result; for practical skill is rarely of general utility or extended application, unless it originates in knowledge. Thus from a knowledge of the science of the tissues with which the dentist comes in contact, he derives the art of producing in them health or disease. The M. D. or D. D. S., in order to do the greatest amount of good to their fellow-men, and arrive at the acme of fame, must possess learning, skill, experience and refinement. They should always be ready to improve any opportunity to gain knowledge which can be made useful to themselves or their patients. All will be ready enough to admit that, the more we know of any and all organs of the human subject, the easier it will be for us to meet the formidable array of complicated diseases which may be presented. Those who are "too smart to learn any thing new" in the unbounded domain of medicine or surgery, should at once be permitted to pass from this "mundane sphere" and enter a higher one (should it be so directed), where their immaterial spirits shall hope to have boundless room for expansion. Many have the possibilities of greatness latent within them, but they keep those innate powers hidden and dormant at the expense of their own higher development, and the sacrifice of those who seek their advice. It would be better to retire, should the time come when you can not command a sufficient recompense to enable you to give your time and very best skill to your patients; for it is only in that way you will be able to progress onward, and conscientiously discharge your duty. D. D. S., if you would succeed well in your practice, you must have much general information which an M. D. possesses. You are required to understand, to a greater or less degree, the workings of the whole human system, and the more you know of its different functions, the better you will be able to diagnose correctly the diseases with which you come in contact; thus from your specialty, and the supposition that you are learned in these matters, it becomes your duty to yourself and patients to stud y and develop your powers in such *general* branches of medicine as an M. D. is obliged to go through, before he can have the right to administer to diseased humanity. Both England and Prussia have given us an example worthy of imitation in this respect, as they require the dental student to attend the same lectures and be examined by the same board of censors as though he were to be a general surgeon. This, with the *special* study which a dentist must have, places him in a just position in his profession, and in a proper light before his patients. Without these acquirements he has not done "his highest," neither has he developed by brain and hand work those powers which ought to receive the culminating stroke, for only thus will he be able to withstand the severe test of time. Now we ask, is it any more than just that an M. D. should be required to have *some* of the general knowledge of a D. D. S.? Would it not be well for him to have a *fair* comprehension of the teeth and their diseases? M. D., remember that in the teeth you are dealing with vitalized structures, supplied with nerves, veins, arteries, and that through these nerves they are connected in close sympathy with all parts of the system. For the sake of your own reputation, study the teeth and their surrounding tissues; for the good of your patients learn to relieve an aching tooth without extracting; save the precious organs, and add in coming years to your own fame, and the health, comfort and beauty of those who seek your counsel. We solemnly protest against the wholesale and indiscriminate sacrifice of these innocents. Do not follow the miserable habit of former ages, and extract any tooth that happens to cause pain, but keep in mind that when the permanent teeth are removed they do not appear again; nature does not supply the terrible chasm you have made, as she does in many other parts of the body, but once gone, forever gone. Always have a good reason for extraction, something more than because you are asked to do so. Do not utterly disregard your moral duty and responsibility by destroying all the teeth you can, thinking that the greater number you exterminate the more glorious has been your victory. Have a higher conception of the demands of your profession, knowing that the more teeth you are instru mental in saving the greater the good you have done. Look around you and see who are the most successful physicians. Are they not those who have made the teeth somewhat of a study,and when called to a case, examine them carefully, should there be the least cause to suspect their implication? Take, for example, a case of facial neuralgia, a most painful affection of the fifth pair of nerves, where the pain is generally located over the orbit, in the temple, cheek, mouth, jaws and teeth; now show us the M. D. who diagnoses these cases properly, and cures the greatest number, and we will show you one who does not fail to examine closely the mouth and teeth. For he knows that pericementitis, arising from whatever cause, produces a most painful type of this disease; also that carious teeth are among the most prominent causes, and should receive his very first attention, for almost invariably he will find them or their contiguous tissues in an unhealthy condition.

We append the following cases: Mr. ——, aged 35, had severe pain in right cheek, orbit and ear; had been treated by an M. D. for four or five months for neuralgia, but obtained no relief. A D. D. S. relieved him in one moment by removing the root of a superior third molar.

Miss ——, aged 13, had an abscess situated upon the neck about twohes below the angle of the inferior maxilla, had been there some months, and was twice lanced by the family physician. Entirely cured by the removal of an inferior molar much decayed and ulcerated. Thus might we continue with similar cases to show the practicability of the above article, which has been written hoping to bring the M. D. and D. D. S. into a closer relationship of brotherly harmony. Let each of us always be ready to assist the other in consultations and operations, and our word for it, both will be benefitted. Do not regard any with jealousy, or look upon them as rivals, but know and remember that you are working together for the common good of human kind, and with such friendly feelings in your hearts, success will in time surely crown your efforts, as "he most lives, who thinks most, feels noblest, acts the best."

